# Hard Study

**Published:** 1856-02

**Authors:** 


### HARD STUDY

Hurts nobody, but hard eating does. It is a very common thing to attribute the premature disability or death of students and eminent men to too close application to their studies. It has now become to be a generally admitted truth, that "hard study," as it is called, endangers life. It is a mischievous error that severe mental application undermines health. Unthinking people will dismiss this with the exclamation of "that's all stuff," or something equally conclusive. To those who search after truth in the love of it we wish to offer some suggestions.

Many German scholars have studied for a life-time for sixteen hours out of the twenty-four, and a very large number from twelve to fifteen hours, lived in comparative health, and died beyond the sixties.

One of the most sterling of living minds, PROF. SILLIMAN, the elder, is now in mid-winter, travelling through the country, at the age of nearly eighty years, and in good health, delivering geological lectures, living mentally on the hard food of rocks, iron, iridium, and the like. Another strong example of the truth that health and hard study are not incompatible, is found in the great Missourian, THOMAS H. BENTON, now past the three-score and ten, and in the enjoyment of vigorous health; a more severe student than he has been, and is now, the American public does not know. Dr. Charles Caldwell, our honored preceptor, lived beyond the eighties, with high bodily health, remarkable physical vigor, and mental force scarcely abated, yet, for a great part of his life he studied fifteen hours out of the twenty-four, and at one time gave but five hours to sleep. John Quincy Adams, the old man eloquent, is another equally strong example of our position. All these men, with the venerable Dr. Nott, now more than eighty years old, made the preservation of health a scientific study, and by systematic temperance, neither blind nor spasmodic, secured the prize for which they labored, and with it years, usefulness and, honor. The inculcation of these important truths was precisely the object we had in view, in the projection of this *Journal*, with the more immediate practical application to the clergy of this country, whom we see daily disabled or dying, scores of years before their time, not as is uniformly and benevolently stated, from their "arduous labors," but by a persistent and inexcusable ignorance of the laws of life and health, and a wicked neglect of them. We use this strong language purposely, for ignorance of duty to their own bodies is no more excusable than ignorance of duty to their own souls, for upon both classes of duty the lights brightly shine, full bright enough for all practical purposes—the lights of nature, of science, of experience and of grace. How much of the hard, intolerant theology of the times was concocted, and is perpetuated by dyspeptic stomachs, reflecting men can readily conjecture. We do not with malice aforethought indite hard things against a class of men so good, able, so useful, as the American clergy are, nor is it any gratification; but we feel that they need to be sharply spoken to; their habit is dictation, and there is none to dictate to them. We take it upon ourselves to guard and guide the shepherds. We would like to say much more on this subject, but long articles are neither read nor copied, and by many, a long cigar or a long *quid* of tobacco would be preferred. For the present, therefore, we content ourselves with the enunciation of the gist of this article. Students and professional men are not so much injured by hard study as by hard eating; nor is severe study for a life-time of itself incompatible with mental and bodily vigor to the full age of threescore years and ten.

